# Impact of physical activity levels on musculoskeletal symptoms and absenteeism of workers of a metallurgical company

**DOI:** 10.47626/1679-4435-2020-572

**Published:** 2021-03-03

**Authors:** Thiago Mattus Ribas, Rosana Macher Teodori, Fabiana Foltran Mescolotto, Maria Imaculada De Lima Montebelo, Silvia Beatriz Serra Baruki, Eli Maria Pazzianotto-Forti

**Affiliations:** 1 Programa de Pós-Graduação em Ciências do Movimento Humano, Universidade Metodista de Piracicaba (Unimep), Piracicaba, SP, Brazil; 2 Universidade Federal de Mato Grosso do Sul (UFMS) - Campus Pantanal, Corumbá, MS, Brazil

**Keywords:** lifestyle, work-related musculoskeletal disorders, repetitive strain injury, absenteeism

## Abstract

**Introduction:**

Physical inactivity is the fourth biggest risk factor for global mortality. In Brazilian metallurgical industries, workers present a high incidence of musculoskeletal symptoms as one of the main causes of absenteeism.

**Objectives:**

To investigate the impact of physical activity levels and leisure-time physical exercise on musculoskeletal symptoms and absenteeism among administrative and production workers of a metallurgical industry.

**Methods:**

This is a transversal study that included 206 workers. We applied the Modified Baecke Questionnaire, leisure-time physical activity and leisure-time physical exercise domains), as well as the Nordic Musculoskeletal Questionnaire regarding symptom occurrence and severity scores (1-4), and compared levels of absenteeism. Our sample was divided into 2 groups: production and office workers.

**Results:**

We observed a significant difference between the groups regarding symptom severity score 3 (p = 0.03) and absenteeism (p = 0.02); the production group presented higher results. There was a correlation between leisure-time physical exercise and absenteeism (r = -0.57, p = 0.01) and between leisure-time physical activity and absenteeism (r = -0.55, p = 0.01) in the production worker group, whereas in the office worker group, leisure-time physical activity and symptom severity score 4 were correlated (r = 0.63, p = 0.02).

**Conclusions:**

Production workers presented higher occurrences of symptom severity score 3 and absenteeism; increased levels of leisure-time physical activity and physical exercise reduced absenteeism. Leisure-time physical activity was correlated with severity score 4 in the office worker group.

## INTRODUCTION

According to the World Health Organization (WHO),^1^ physical inactivity is considered a global epidemic that affects almost 70% of the world’s population. According to Stevens et al.,^2^ physical inactivity is one of the most important public health problems in the 21st century. Worldwide, 1 every 4 adults does not reach the recommended levels of physical activity, and inactivity levels increase along with the countries’ economic development due to changes in modes of transportation, the higher use of technology, and urbanization.^3^ Children and adolescents are recommended 60 minutes of moderate- to vigorous-intensity physical activity daily, while adults (over 18 years old) should have 150 minutes of moderate-intensity physical activity per week.^1^

Considering the length of work tasks and the position in which workers execute them, employees can be at risk of various musculoskeletal symptoms due to low muscle strength and endurance.^4^ This is especially problematic in metallurgical industries, where production happens at large scales and tasks are inherently physically demanding, frequently requiring biomechanically inadequate postures, repetitive movements, and an intense work pace.^5^ Among office jobs, long work hours that involve sitting for long periods can also compromise workers’ musculoskeletal health, but since technology is fundamental in today’s labor market, the intense use of computers has become inevitable.^6^

Diseases of the musculoskeletal system can trigger different degrees of functional incapacity, being considered major occupational health problems. In 2007, the rate of disability retirements related to musculoskeletal disorders of the spine was 30 per 100 thousand workers; these problems were more frequent among men and represented the main cause of disability leading to retirement,^7^ in addition to being one of the main causes of absenteeism, which increases company expenses.^8^

In Great Britain, around 9.5 million working days were lost due to musculoskeletal disorders.^9^ The International Labor Organization^10^ has defined absenteeism as an absence from work that is accepted due to worker’s incapacity, except for a normal pregnancy period or prison time. Thus, absenteeism can be classified into participant, legally compulsory, due to professional pathology, or to illness.^11^

In view of this information and considering that regular physical activity could be associated to a lower occurrence of musculoskeletal symptoms and absenteeism, we hypothesized that workers with higher levels of regular physical activity should present less musculoskeletal symptoms and absenteeism. Therefore, our study is important not only for comprehending the relationship between physical activity levels, musculoskeletal symptoms, and absenteeism, but also as a foundation for future studies on the impact of physical inactivity on companies; this could contribute to suggesting practical interventions to improve the health and quality of life of workers. Altogether, the aim of this study was to investigate the impact of leisure-time physical activity on musculoskeletal symptoms and absenteeism among workers of a metallurgical company.

## METHODS

This is a transversal observational study that included workers of a metallurgical company of the state of São Paulo. Our research was approved by the Research Ethics Committee (2.361.266) according to resolution No. 466/12 of the National Health Council regarding research with human beings.

Our inclusion criteria were workers admitted before December 31, 2016, and aged between 20 and 60 years. Exclusion criteria considered workers that were on leave, provided incomplete or unanswered questionnaires, and those subjected to surgical procedures between January 1 and December 31, 2017.

Our sample included 217 participants who were interviewed during routine visits to the company’s outpatient clinic; these included production and office workers. Our data collection was performed between November 2017 and April 2018. Out of 217 initial workers, 11 did not correspond to the inclusion criteria and were removed from the study; our final sample thus consisted of 206 participants.

The sample was divided in 2 groups, according to work domains - Group P included production workers and Group O comprised office workers - in order to evaluate the impact of physical activity on the following variables: musculoskeletal symptoms (number of occurrences [NO] and severity score [Sev.]), and absenteeism index (AI).

The experimental protocol initially used 2 self-administered questionnaires, with prior explanation by a blinded evaluator regarding the research objectives and interpretation of questionnaire data. The questionnaires aimed to measure the physical activity levels and musculoskeletal symptoms of the participants.

## MODIFIED BAECKE QUESTIONNAIRE (MBQ)^12^

This protocol comprised 16 closed questions considering physical activities along 3 main domains: (1) occupational physical activities (OPA); (2) leisure-time sport activities (LSA); (3) leisure and commuting activities (LCA). We thus determined the scores for each physical activity domain and the sum of these scores was performed according to Baecke et al..^12^ We did not consider OPA as a variable of interest since the differences in energy expenditure between both groups (office and production workers) were too great, and our objective was to evaluate physical activity performed outside of the work environment. Therefore, the level of physical activity considered in our analysis was the sum of LSA and LCA scores (participants were subdivided into quartiles according to total scores, resulting in the following classification: first quartile, physically inactive; second and third quartiles, moderately active; fourth quartile, physically active).

## NORDIC MUSCULOSKELETAL QUESTIONNAIRE (NMQ)^13^

We used the general questionnaire that comprised all anatomical areas, as well as 2 specific sections for the low-back, neck, and shoulder areas. Answers were multiple-choice or binary, regarding the occurrence of symptoms and the most common anatomical areas. The participant should report symptom occurrence considering 3 questions: regarding the last 7 days, the last 12 months, or his/her whole life. After recording the answers for each area of the body, Sev. 1-4 accounted for the amount of reported occurrences: score 1 represented only 1 occurrence in any of the defined periods; 2 represented 2 occurrences, of which 1 was in the last 12 months and the other was in the last 7 days; score 3 meant 2 occurrences, of which one was in the last 12 months and the other, in the last 12 months or in the last 7 days; and 4 indicated 1 occurrence for each of the 3 periods. Subsequently, we processed data regarding absenteeism due to musculoskeletal problems using the formula proposed by Marras:^14^ AI=Nhl/Nph×100, where AI is the absenteeism index, Nhl is the number of hours lost, and Nph in the number of planned hours. Each AI unit, in this company, represented a financial loss of US$ 11.20.

Sample size calculations (r = 0.21, considering α = 0.05 and 80% statistical power) using data from a pilot sample of 100 workers resulted in a sample size of 180 participants. Our data were descriptively evaluated through frequencies, percentages, means, and standard deviations. We used the Kolmogorov-Smirnov test for assessing data normality, and then the Mann-Whitney U test to evaluate differences between variables for each group and for correlations between the LSA and LCA domains. For all other variables, we used the Spearman’s correlation test. Statistical significance was defined by p < 0.05, and analyses were performed using SPSS software, version 22.0.

## RESULTS

Table 1 presents the general characteristics of our sample (sex, anthropometric profile, physical activity level, and NO, per area).

**Table 1:** Sample characteristics

	Total	Group O	Group P
Sample (n)	206	102	104
Sex (M/F)	166/40	70/32	96/8
Anthropometric profile (Mean±SD)			
Age (years)	38.5±8.8	38.5±9.0	38.4±8.6
Height (m)	1.73±0.08	1.72±0.08	1.73±0.08
Weight (Kg)	82.2±14.8	80.1±15.5	84.3±13.8
BMI (Kg/m^2^)	27.4±4.1	26.9±4.0	27.8±4.2
NO, per area [n (%)]			
Neck	74 (13.5)	42 (16.4)	32 (10.5)
Right shoulder	61 (11.1)	31 (12.1)	30 (9.8)
Left shoulder	39 (7.1)	18 (7.0)	21 (6.9)
Right elbow	15 (2.7)	4 (1.6)	11 (3.6)
Left elbow	14 (2.6)	2 (0.8)	12 (3.9)
Right forearm	16 (2.9)	2 (0.8)	14 (4.6)
Left forearm	11 (2.0)	2 (0.8)	9 (3.0)
Right wrist/hand/fingers	34 (6.2)	15 (5.9)	19 (6.2)
Left wrist/hand/fingers	26 (4.7)	10 (3.9)	16 (5.2)
Upper/middle back	42 (7.7)	24 (9.4)	18 (5.9)
Lower back	88 (16.7)	47 (18.4)	41 (13.4)
Hips and thighs	24 (4.4)	12 (4.7)	12 (3.9)
Knees	70 (12.8)	35 (13.7)	35 (11.5)
Ankles and feet	34 (6.2)	12 (4.7)	22 (7.2)

M/F = male/female; SD = standard deviation; BMI = body mass index; NO = number of musculoskeletal symptom occurrences.

Most workers were male (166, of which 70 belonged to Group E and 96 to group P) and our sample was equally distributed between office and production workers (102 and 104, respectively). Mean age was 38.5 years and mean BMI was 27.4, indicating overweight; both aspects had little variation. The areas of the body that presented the most NO were the lower back, neck, knees, and right shoulder, of which low-back symptoms were present in 88 participants (18.4% of Group O and 13.4% of Group P). Within Group O, the second leading area in NO was the neck (16.4%), followed by the knees (13.1%), and right shoulder (12.1%). In Group P, the second leading body part in NO were the knees (11.5%), followed by the neck (10.5%), and right shoulder (9.8%).

Table 2 shows comparisons between variables LSA, LCA, NO, Sev. (1-4), and AI (in hours), calculated according to Marras.^14^

**Table 2:** Comparisons between variables

Variables	Group O (Mean±SD)	Group P (Mean±SD)	p
LSA	2.82±0.61	2.76±0.69	0.41
LCA	8.46±2.00	8.15±2.07	0.45
NO	3.27±2.04	3.74±2.88	0.68
Sev.			
1	2.23±1.43	1.76±0.99	0.12
2	2.06±1.19	2.88±2.15	0.13
3	1.47±1.26	2.24±1.56	0.03^*^
4	1.50±0.86	2.80±2.80	0.10
AI (h)	2.82±1.56	97.55±223.12	0.02^*^

AI (h) = absenteeism index, in hours; LSA = leisure-time sport activities; LCA = leisure and commuting activities; NO = number of musculoskeletal symptom occurrences; Sev. = severity score; SD = standard deviation.

* Significant result (p < 0.05), Mann-Whitney U test.

Significant differences were observed between Groups O and P for Sev. 3 and AI (p = 0.03 and 0.02, respectively), where Group P had higher results.

Tables 3 and 4 present the results of correlations (in the whole sample and each of the groups) between physical activity domains and the following variables: NO, Sev., and AI. Table 3 presents results and their correlations with the LSA domain.

**Table 3:** Correlations between the leisure-time sport activities domain and results of number of musculoskeletal symptom occurrences, severity score, absenteeism index

	LSA			
	Group O		Group P	
	r	p	r	p
NO	0.06	0.58	-0.10	0.40
Sev.				
1	0.04	0.77	-0.21	0.22
2	0.02	0.92	0.07	0.65
3	0.22	0.38	0.09	0.72
4	0.21	0.35	0.20	0.33
AI (h)	0.50	0.67	-0.57^*^	0.01^†^

AI (h) = absenteeism index, in hours; LSA = leisure-time sport activities; NO = number of musculoskeletal symptom occurrences; r = coefficient of correlation; p = significance; Sev. = severity score.

* Correlation between LSA and absenteeism in Group P: moderately negative.

† Statistical significance: p < 0.05.

**Table 4:** Correlations between the leisure and commuting activities domain and number of musculoskeletal symptoms, severity score, and absenteeism index

	LCA			
	Group O		Group P	
	r	p	r	p
NO	-0.01	0.90	-0.18	0.11
Sev.				
1	-0.01	0.91	-0.18	0.31
2	-0.26	0.13	-0.06	0.72
3	0.14	0.57	-0.06	0.83
4	0.63^*^	0.02^†^	0.06	0.79
AI (h)	0.50	0.67	-0.55^*^	0.01^†^

AI (h) = absenteeism index, in hours.LCA = leisure and commuting activities; NO = number of musculoskeletal symptoms occurrences; r = coefficient of correlation; p = significance; Sev. = severity score.

* Correlation between LCA and Sev. 4 in Group O: strongly positive; correlation between LCA and AI in Group P: moderately negative.

† Statistical significance: p < 0.05.

Results presented in Table 3 indicate an inverse relationship between the LSA domain and the AI variable in Group P. This means that a more intense and consistent level of leisure-time physical activity implies in lower levels of absenteeism, which is an important result for the company.

Table 4 presents the obtained results and their correlations with the LCA domain.

The results shown in Table 4 also indicate a relationship between the LCA domain and AI in Group P, similarly to that indicated by Table 3 regarding the LSA domain. This goes to show that no matter the type of physical activity or exercise performed during the workers’ leisure time, they presented a direct relationship with a reduction in absenteeism.

Table 4 also indicated a strong positive correlation between the LCA domain and Sev. 4 in Group E, indicating a significant association between these variables (as one of them increases, the other also presents higher values).

## DISCUSSION

Our hypothesis that workers with higher levels of regular physical activity should present less musculoskeletal symptoms and absenteeism^15^ was partially confirmed, since data indicated that physical activity is associated to lower AIs. However, regarding the LCA domain, we did not observe an overall reduction in musculoskeletal symptoms.

Results showed a statistical difference between Groups O and P regarding variables Sev. 3 and AI. Higher results of Sev. 3 in Group P indicated a higher severity and complexity of the musculoskeletal symptoms reported by production workers; this corroborates the higher absenteeism rate in this group. These differences may be related to the physical demands of the job, which require movements of spinal, elbow, neck, and lower limb flexion, shoulder flexion and abduction, and wrist bending. On the other hand, workers in Group O faced lower demands of physical load and movements of the lower limbs and torso.

Our results also indicated that the LCA domain did not exert a definitive protective effect against musculoskeletal symptoms. One possible reason is that this type of physical activity is not planned and reaches lower intensities, thus not always promoting adequate body adaptation. Nevertheless, both LCA and LSA domains significatively impacted absenteeism, displaying inverse relationships with this variable.

While there were no significant differences in NO between groups, the number of participants that reported Sev. 3 was higher in Group P and there was a positive correlation between LCA and Sev. 4 in Group O. Overall, these results indicate that light-intensity physical activity such as walking, for example, was positively correlated with worse musculoskeletal symptoms on Group O, therefore not providing a protective effect.^16^

Still regarding the LCA domain, even high levels of physical activity were not enough to promote body adaptation as a protective factor against musculoskeletal symptoms, since in Group O, this domain was positively correlated to Sev. 3 (indicating withdrawal from activities of daily living). Therefore, among people performing sedentary work, leisure or commuting physical activities do not seem to have a protective effect on the musculoskeletal system, highlighting the need for more intense and controlled physical exercise programs in order to promote positive body adaptations for musculoskeletal health. Due to a high demand for cyclic muscle contractions and articular movements during commuting activities, the lack of physical conditioning for strength and flexibility could be another aggravating factor. This was reported by a study that evaluated combined training performed twice a week in a systematic manner, indicating a protective effect on the musculoskeletal system related to work.^16^ Moreover, vigorous-intensity physical exercise performed 3 days a week has been shown to reduce low-back pain, as well as neck and shoulder pain.^17^ Therefore, workers should adopt a healthy lifestyle along with physical exercise, performed at least in moderate intensity (3 to 5.9 metabolic equivalents [MET]/h) most days of the week.^18^

High occurrences of shoulder and neck symptoms in Group O may reflect the high rate of physical inactivity and characteristics of office work. Sitting for more than 6 hours in a kyphotic angle increases pressure on the intervertebral disc nuclei in 85%, leading to degeneration of these bone structures.^19^ In lordotic posture, there is less pressure inside intervertebral discs when compared to kyphotic posture, although with less activity of the spine extensor muscles and less tension on the posterior ligament;^20^ this way, physical inactivity weakens the paravertebral and abdominal muscles and favors postural dysfunction.

Regarding Group P, the physical and biomechanical demands of work worsen the workers’ musculoskeletal problems. The second leading symptom occurrence being on the knees, with higher Sev. (3 and 4), can be explained by the higher physical demands of this sector, considering displacements, long periods standing and/or squatting, in addition to climbing and descending machines and elevated areas. Considering the third and fourth areas with the most symptom occurrences (neck and shoulders) problems could arise due to reflexes of the cervical spine and high demands of shoulder flexing in the tasks performed by these workers. A study with production line workers showed a high prevalence of musculoskeletal disorders in the lower back, shoulders, and cervical spine, being related to inadequate posturing.^21^ Workers that are exposed to repetitive movements, inadequate posturing, and little recovery time between workdays present a risk of impairment of their work capacity due to fatigue and musculoskeletal disorders.^22^ Impairments of muscle function regarding joint movements observed in workers with chronic pain predispose them to increased muscle and joint strain and contribute directly to lower muscle endurance, negatively impacting work.^17^

In this context, the lack of specific physical exercises both in the workplace and during leisure time can be definitive for the worsening of musculoskeletal health. Physical exercise has thus been used as a tool for prevention and treatment of occupational diseases, showing promising results regarding the reduction of musculoskeletal symptoms.^23^ Moreover, systematic and regular physical activity has shown great benefits to the health and well-being of workers.^24-26^

A review by Oliveira et al.^27^ showed that intense physical exercise programs (over 7 MET/h) could result in clinically important effects for reducing low-back pain; however, no benefits were observed in workers with acute and subacute pain. Our results, along with other reports in the literature, highlight the importance of physical exercise outside of the workplace (mainly when performed in a more intense and systematic manner) as an essential protective factor of workers’ functional capacity; it could prevent or reduce the occurrence of medium- and long-term musculoskeletal symptoms and their severity.

Our results indicated higher levels of absenteeism and physical activity in Group P. LSA and LCA domains presented moderately negative correlations with absenteeism, suggesting that the higher the physical activity levels, regardless of the domain, the lower the absenteeism levels and the higher the workers’ productivity. Group P showed a higher tendency of absenteeism, namely among workers with the lowest levels of regular physical activity. This shows the impact of physical activity levels on this variable and could be related to the positive impact exerted by good physical conditioning in workers that perform physically demanding tasks.

Group O displayed lower absenteeism levels, which could be explained by the lower physical demands of this type of work. Musculoskeletal conditions such as the association between Sev. 4 and LCA, in this situation, would not represent aggravating factors and the rest time between workdays could be enough to provide relief to the workers’ musculoskeletal symptoms, thus reducing absenteeism. Schwatka et al.^24^ performed a study involving 763 office workers; those that participated in a physical exercise program presented lower absenteeism rates throughout 12 months when compared to those who did not participate in the program. Hogsbro et al.^28^ reported that physically inactive workers had a 27% higher number of sick leaves and higher levels of absenteeism when compared to those who were physically active. A systematic review performed by Bueno et al.^29^ showed that regular vigorous-intensity physical activity (over 6 MET/h) was key and effective in reducing absenteeism. In addition, evidences show that there is an inverse correlation between regular physical activity levels and absenteeism.^30^

Altogether, these data suggest that employing and stimulating health care programs that involve regular physical exercises could be an excellent strategy to be adopted by companies in order to reduce musculoskeletal problems and absenteeism, thus improving workers’ health, quality of life, and productivity.

Some limitations of the present study include the fact that we did not use precision tools (such as a digital accelerometer and electromyography, which are commonly used in intervention studies) to evaluate physical activity levels and musculoskeletal symptoms. We did not divide the analysis of production workers according to their tasks, and did not consider the types of physical activity performed by the physically active workers during their leisure time. Nevertheless, the results presented by this study highlight the need for initiatives that promote workers’ health in the metallurgy sector, thus reinforcing the importance of other studies in this area.

## CONCLUSIONS

Workers of Group P presented higher Sev. 3 scores and absenteeism levels. Higher levels of regular physical activity (regardless of the domain) reduced absenteeism in the studied population, while physical activity represented by the LCA domain had a positive correlation with Sev. 4 in group O.

## References

[1] World Health Organization (2011). Global status report noncommunicable diseases 2010.

[2] Stevens RJ, Roddam AW, Spencer EA, Pirie KL, Reeves GK, Green J (2009). Factors associated with incident and fatal pancreatic cancer in a cohort of middle-aged women. Int J Cancer.

[3] World Health Organization (2018). Global action plan on physical activity 2018-2030: more active people for a healthier world.

[4] Assunção AA, Vilela LVO (2009). Lesões por esforços repetitivos: guia para profissionais de saúde.

[5] Barlach L, Limongi-França AC, Malvezzi S (2008). O conceito de resiliência aplicado ao trabalho nas organizações. Interam J Psychol.

[6] Kaliniene G, Ustinaviciene R, Skemiene L, Vaiciulis V, Vasilavicius P (2016). Associations between musculoskeletal pain and work-related factors among public service sector computer workers in Kaunas County, Lithuania. BMC Musculoskelet Disord.

[7] Brasil, Ministério da Saúde, Secretaria de Políticas de Saúde (2000). Protocolo de investigação, diagnóstico, tratamento e prevenção de lesões por esforços repetitivos/distúrbios osteomusculares relacionados ao trabalho.

[8] Filho Meziat N, Azevedo e Silva G (2011). Invalidez por dor nas costas entre segurados da Previdência Social do Brasil. Rev Saúde Pública.

[9] Health and Safety Executive (2015). Work-related Musculoskeletal Disorders (WRMSDs) Statistics, Great Britain, 2015.

[10] Organización Internacional Del Trabajo, Organización Internacional Del Trabajo (1989). Absentismo: causa y control. Enciclopedia de Salud y Seguridad en el Trabajo.

[11] Quick TC, Lapertosa JB (1982). Análise do absenteísmo em uma usina siderúrgica. Rev Bras Saúde Ocup.

[12] Baecke JA, Burema J, Frijters JE (1982). A short questionnaire for the measurement of habitual physical activity in epidemiological studies. Am J Clin Nutr.

[13] Kuorinka I, Jonsson B, Kilbom A, Vinterberg H, Biering-Sørensen F, Andersson G (1987). Standardised Nordic questionnaires for the analysis of musculoskeletal symptoms. Appl Ergon.

[14] Marras JP (2000). Administração de recursos humanos: do operacional ao estratégico.

[15] Oliveira MM, Andrade SSCA, Souza CAV, Ponte JN, Szwarcwald CL, Malta DC (2015). Problema crônico de coluna e diagnóstico de distúrbios osteomusculares relacionados ao trabalho (DORT) autorreferidos no Brasil: Pesquisa Nacional de Saúde, 2013. Epidemiol Serv Saúde.

[16] Machado AR (2013). As perturbações músculo-esqueléticas no trabalho em saúde: O caso de uma Unidade de Cuidados Continuados Integrados de Média Duração e Reabilitação.

[17] Sundstrup E, Jakobsen MD, Brandt M, Jay K, Aagaard P, Andersen LL (2016). Strength Training Improves Fatigue Resistance and Self-Rated Health in Workers with Chronic Pain: A Randomized Controlled Trial. Biomed Res Int.

[18] Agência Europeia para a Segurança e Saúde no Trabalho (2007). Introdução às lesões músculo-esqueléticas.

[19] Marques NR, Hallal CZ, Gonçalves M (2010). Características biomecânicas, ergonômicas e clínicas da postura sentada: uma revisão. Fisioter Pesqui.

[20] Silva JN, Neto RC (2016). Prevalência de dor lombar em pessoas que trabalham na postura sentada. Rev UNILUS Ens Pesqui.

[21] Shahriyari M, Afshari D, Latifi SM (2018). Physical workload and musculoskeletal disorders in back, shoulders and neck among welders. Int J Occup Saf Ergon.

[22] Van Rijn RM, Huisstede BM, Koes BW, Burdorf A (2010). Associations between work-related factors and specific disorders of the shoulder--a systematic review of the literature. Scand J Work Environ Health.

[23] Sundstrup E, Jakobsen MD, Andersen CH, Jay K, Persson, Aagard P (2014). Effect of two contrasting interventions on upper limb chronic pain and disability: a randomized controlled trial. Pain Physician.

[24] Schwatka VN, Smith D, Weitzenkamp D, Atherly A, Dally JM, Brockbank SVC (2018). The Impact of worksite wellness programs by size of business: A 3-year longitudinal study of participation, health benefits, absenteeism, and presenteeism. Ann Work Expo Health.

[25] Malinska M (2017). Effectiveness of physical activity intervention at workplace. Med Pr.

[26] Zica MM, Morbeck NBM, Quaresma FRP, Brito EN, Adami F, Maciel ES (2015). Avaliação do nível de atividade física, composição corporal, percepção da qualidade de vida e presença de dor em funcionários de uma empresa. Rev Cereus.

[27] Oliveira VC, Jardim GC, Kamper SJ (2018). Do physical conditioning programmes reduce work absenteeism related to back pain: (PEDro synthesis). Br J Sports Med.

[28] Høgsbro C, Davidsen M, Sørensen J (2018). Long-term sickness absence from work due to physical inactivity: A registry-based study. Scand J Public Health.

[29] Bueno RL, Mallén JAC, Vallejo NG (2018). La actividad física como herramienta para reducir el absenteísmo laboral debido a enfermedad em trabajadores sedentarios: Una revisión sistemática. Rev Esp Salud Pública.

[30] Kerner I, Rakovac M, Lazinica B (2017). Leisure-time physical activity and absenteeism. Arh Hig Rada Toksikol.

